# Prenatal infant feeding intentions and actual feeding practices during the first six months postpartum in rural Rwanda: a qualitative, longitudinal cohort study

**DOI:** 10.1186/s13006-020-00275-y

**Published:** 2020-04-17

**Authors:** Jeanine Ahishakiye, Laura Bouwman, Inge D. Brouwer, Lenneke Vaandrager, Maria Koelen

**Affiliations:** 1 Health and Society Chair Group, Wageningen University, Wageningen, The Netherlands; 2 Human Nutrition and Dietetics department, College of Medicine and Health Sciences, University of Rwanda, Kigali, Rwanda; 3 Division of Human Nutrition and Health, Wageningen University, Wageningen, The Netherlands

**Keywords:** Breastfeeding intentions, Exclusive breastfeeding, Qualitative research, Child nutrition, Barriers, Facilitators

## Abstract

**Background:**

Exclusive breastfeeding (EBF) is advocated by the WHO for the first 6 months. In Rwanda, the percentage of infants who are exclusively breastfed decreases from 94% among infants aged 0–1 month to 81% among those aged 4–5 months. Little is known about what influences mothers’ breastfeeding practices. This study aimed to gain insights into expectant mothers’ prenatal feeding intentions, the underlying reasons, actual practices after birth, and factors facilitating or impeding EBF for the first 6 months of a child’s life in Muhanga District, Rwanda.

**Methods:**

This qualitative longitudinal study, conducted between December 2016 and October 2017 as part of a larger study, recruited a purposive sample of 39 pregnant women attending prenatal consultations during their last trimester in two rural health centers. Women were interviewed during pregnancy, within the first week after birth and at 4 and 6 months postpartum to explore intentions, actual practices, critical transition points, and facilitating or impeding factors. Interviews were recorded, transcribed verbatim, and analyzed thematically.

**Results:**

Of the 39 participants, 38 intended to breastfeed within the first hour after birth, and 32 intended to breastfeed exclusively for the first 6 months. In practice, 34 initiated breastfeeding within the first hour, and 12 breastfed exclusively for 6 months. Impeding factors include perceived breastmilk insufficiency, pressure from family members, past experiences, mothers’ concerns over their infants’ health, mothers’ heavy workload, poverty and food insecurity. Factors facilitating early initiation and EBF include mothers’ awareness of EBF’s advantages, confidence in their breastfeeding ability, and support from health professionals and family members.

**Conclusion:**

Despite participants’ intentions about breastfeeding, there was a gap between intentions and actual practices. An interplay of barriers at individual, group and societal levels impeded women from EBF for the first 6 months. EBF promotion interventions should consider supporting and equipping breastfeeding mothers with skills to deal with perceived breastmilk insufficiency and to recognize the true signs of baby hunger cues. Furthermore, important influential family and community members should be targeted to support mothers to breastfeed. Interventions that consider addressing the issue of poverty-driven food insecurity should not be overlooked either.

## Background

Breastfeeding is the first fundamental right of the child. The World Health Organization (WHO) and UNICEF recommend that newborns should be breastfed within 1 h after birth, be exclusively breastfed during the first 6 months of life, and continue breastfeeding until 24 months of age with appropriate complementary feeding initiated at 6 months [1]. Early initiation of breastfeeding within the first hour after birth is associated with significantly reduced neonatal mortality [2]. Exclusive breastfeeding (EBF) for the first 6 months reduces the risk of many diseases, mainly gastrointestinal, respiratory infections, and chronic diseases affecting infants [3–5]. It also promotes sensory and cognitive development in infants [5–7]. As noted by Dewey and Huffman [8], a combination of factors including lack of EBF in the first 6 months exposes older children to stunting. Lack of EBF for the first 6 months has been found to be associated with increased risk of infections such as diarrhea, especially in developing countries, which further contribute to malnutrition and poor growth [9]. Despite the evidence supporting the importance of EBF for child health, breastfeeding practices remain suboptimal worldwide, especially in low- and middle-income countries, where only 38% of infants aged 0 to 6 months are exclusively breastfed [5], among which Rwanda is classified. The determinants of breastfeeding practices operate at multiple levels and affect breastfeeding intentions and behaviors over time. Personal factors such as knowledge about the benefits of breastmilk [10], structural factors, and undesirable socio-cultural beliefs and misconceptions prevailing in the community [11–13] and amongst healthcare providers [14] are reported to influence breastfeeding practices. However, the way in which the factors influence EBF practices are contextual and differ from one setting to the next, necessitating setting-specific data.

The Rwanda National Food and Nutrition Strategic Plan 2013–2018 promotes EBF, encouraging mothers to initiate breastfeeding within the first hour of birth and to continue EBF for 6 months, after which complementary feeding is introduced to work alongside breastfeeding for at least 2 years [15]. Data from the Rwanda Demographic and Health Survey (RDHS) 2014–2015 show that 94% of infants aged 0–1 month are exclusively breastfed whereas this is reduced to 81% for infants aged 4–5 months [16].

Although these estimates are available, no insight is provided on the “why” and “how” of the status quo, including “what” and “who” influences mothers’ breastfeeding decisions and practices. This information is important for informing the design of context-specific and targeted programs and policy interventions that will effectively improve health and nutrition outcomes for children in Rwanda. The aim of this study was to gain insights, from a longitudinal qualitative study, into prenatal maternal infant feeding intentions, the reasons for these intentions, actual practices, and factors that facilitate and impede recommended infant feeding practices during the first 6 months of a child’s life in a rural district of Rwanda.

## Methods

### Study design

The study adopted a longitudinal qualitative design to explore prenatal maternal feeding intentions, actual feeding practices from birth to 6 months of age, and factors facilitating or impeding recommended practices. Four interview points were used. To explore breastfeeding intention, interviews were conducted during pregnancy. To explore actual practices, our study adopted the WHO and UNICEF recommendations of initiating breastfeeding within 1 h of birth and to continue with EBF for 6 months [1]. Interviews were conducted within the first week after birth and at 4 and 6 months postpartum, indicating the critical transition points (periods at which changes in feeding practices occur), to explore feeding practices and factors facilitating or impeding recommended practices.

The study, undertaken between December 2016 and October 2017, was conducted within the framework of a larger longitudinal study aiming to unravel the postnatal determinants of stunting in the area, with a total study population of 190 mother–child pairs. Both studies were undertaken at the same time, but with different orientations and using different theory-based methods. The larger quantitative study had a nutritional-physiological orientation to investigate the determinants of child growth whereas this current study had a social orientation toward understanding infant and young child feeding practices. There was no difference in treatment between the children in the study and the other children. All children were measured monthly by Community Health Workers (CHWs), who gave feedback to the mothers. The only difference is that the children included in the study were also measured for anthropometry by the researchers.

### Theoretical basis of the study

The Theory of Planned Behavior (TBP) guided the exploration of mothers’ decisions and practices [17]. The TPB focuses on the psychological factors influencing behaviors. It posits that the immediate antecedent of a behavior is the person’s intention to perform the behavior. The intention, in turn, is influenced by the attitude toward the behavior, subjective norm, and perceived behavioral control. Attitude toward the behavior refers to the overall evaluation, either positive or negative, of performing the behavior. Subjective norm reflects the perceived social pressures to perform or not to perform the behavior. Perceived behavior control, also called self-efficacy, refers to the perceived ease or difficulty of performing the behavior.

### Study setting

The study was conducted in the catchment area of two rural health center facilities (Rutobwe and Buramba) in Muhanga District, approximately 49 km south of Kigali City. The main activity is agriculture (practiced by 76% of households), and it is also the main source of income, although 90% of total production is for home consumption. Although 39.1% of the Rwandan population was below the poverty line as per the 2013–2014 Household Living Conditions Survey [18], Muhanga District was one of the best performers because it had reduced the poverty headcount from 53.6% in 2010 to 30.5% within the following 3 years. However, the 2014–2015 RDHS found that 41.6% of under-five children were stunted in Muhanga District; this is higher than the national average of 38% [16]. EBF is promoted countrywide, including in the study setting. At the health center facility level, mothers receive health and nutrition education including awareness raising on WHO recommended infant feeding practices from nurses during antenatal and postnatal care visits. The same awareness raising continues at the community level in every village (the lowest administrative unit) by CHWs during the antenatal and postnatal periods. Each village has: a) a pair of general CHWs who are responsible for community health, nutrition, and HIV/AIDS prevention; b) a maternal health worker, who manages infant, prenatal, and postnatal maternity care; and c) a CHW in charge of social affairs dedicated to addressing the wellbeing of individuals and the community.

### Study population and sampling procedure

Women in the last trimester of pregnancy, visiting governmental health centers in Buramba and Rutobwe, were approached while queuing for prenatal care and were briefed about the study objectives. A total of 60 pregnant mothers out of 190 enrolled in the larger quantitative study were willing to be involved in our study. Recruitment stopped after enrolling 39 women in the study as data saturation had been reached. Inclusion criteria for the study consisted of being pregnant in the last trimester of pregnancy with no serious obstetrical conditions. Women who did not plan to reside in the area with the baby for the first year were excluded from the study.

### Data collection and measurements

Open-ended in-depth interview guides were used to explore prenatal feeding intentions, actual feeding practices at critical transition points, and factors facilitating or impeding recommended and/or intended practices from birth to 6 months postpartum. During enrolment, information was collected about participants’ socioeconomic and demographic characteristics. In addition, women were requested to state their infant feeding plans from birth to 6 months of age. Participants provided the expected delivery dates and were contacted regularly until the baby was born. All 39 mothers delivered in health centers.

The interview questions were developed based on the TPB constructs [17]. To align with the qualitative nature of the study, prenatal intention, attitude, subjective norm, and perceived behavior control were assessed using self-reported open-ended questions instead of scales. Infant feeding intention was assessed by asking participants how they intended to feed their expected babies after birth, during the first 3 days after delivery, during the first month, and between 1 and 6 months of age. Attitude toward breastfeeding was assessed by asking women about their belief in the advantage or disadvantage of breastfeeding the baby within the first hour after birth and of EBF for the first 6 months. The questions on the subjective norm construct looked more broadly at the women’s perceived social pressure to perform or not perform EBF. The perceived behavior control questions related to factors that women perceived would help them to breastfeed their baby within the first hour after birth and to breastfeed exclusively for the first 6 months, as well as factors that would stop them from doing so. During the postpartum period, in-depth interviews were conducted in participants’ homes. Follow-up interviews included open-ended questions that focused on exploring actual feeding practices and on obtaining a deeper understanding of factors facilitating or impeding the WHO-recommended practices.

The interview guides were piloted with both prenatal and postnatal mothers of young children attending nutrition rehabilitation at the health centers, and adjustments were made accordingly. Table 1 summarizes the content of the interview guides. All interviews lasted between 30 and 60 min and were conducted by the first author with the help of a trained field assistant in the local language (Kinyarwanda). The field assistant was recruited in a competitive process. She had a bachelor’s degree and some experience in qualitative data collection.

**Table 1:** Content of the interview guides

Period	Questions
Pregnancy	- How do you intend to feed your expected baby: (You walk through the baby’s ages and discuss how they intend to feed the baby and with what) • After birth? • During the first three days after delivery? • During the first month (after the first three days) • Between 1 and 6 months of age? - How long after birth do you intend to breastfeed the baby for the first time? - How long do you intend to feed your expected baby with breast milk only? - Why did you think you would feed your baby this way?
First week	- For this new baby (the name of the baby), how long after birth was she/he breastfed? - [If less than one hour] What do you believe are the advantages of breastfeeding the baby within the first hour after birth? - What factors or circumstances enabled you to breastfeed the baby within the first hour after birth? - [If more than one hour] What factors or circumstances made it difficult for you to breastfeed the baby within the first hour after birth? - Was the baby given (by you or anyone else) anything to eat/drink before he/she was breastfed for the first time? - [If yes] What was given to the baby? - Why was it given to her/him? [Ask for each food/drink that was given to the baby] - Who advised you to give this to the baby? [Ask for each food/drink that was given to the baby] - At the moment, from birth till now, has the baby been given anything to eat or drink other than breastmilk? - What was given to the baby other than breast milk? - What was the reason that triggered you to offer your baby (drink or/food item)? - Who advised you to do that?
4 months	Now let’s talk about how the baby was fed after these few days. Please think back from the first week through the first few months: - Are you currently breastfeeding the baby? If yes, how often do you breastfeed? - Do you breastfeed 1) on a fixed schedule, or 2) each time the baby asks to be fed, or 3) depends on the mother’s availability? If not, at what age of the child did you discontinue breastfeeding? Why did you stop breastfeeding the baby? - What type of food was given to the baby; if any, what were the reasons that triggered you to offer your baby (drink or food mentioned)? - How did you learn about that? Did you learn it from someone? - From the last visit, in the first week postpartum, till now, did you make some changes in the ways you feed your baby? If yes, what did you change? - Did you have any problem or challenge (internal or external) impeding the ideal breastfeeding practices for your child? For every problem, probe to talk more about the problem. - What elements do you believe help you to overcome those challenges? Is it something inside you (physical, mental, or spiritual) or something outside yourself (social and physical context)?
6 months	Now let’s talk about how the baby was fed after these few months. From the last visit in the fourth month till now, did you make some changes in the ways you feed your baby? If yes, what did you change? Probing questions: - Are you currently breastfeeding the baby? If yes, how often do you breastfeed? - Do you breastfeed 1) on a fixed schedule, or 2) each time the baby asks to be fed, or 3) depends on the mother’s availability? If not, at what age of the child did you discontinue breastfeeding? Why did you stop breastfeeding the baby? - Have you given any food/drink to your baby in addition to breastmilk? - If yes, what was the first food given to your baby? - How old was your baby when you gave him/her this particular food or drink? If food was introduced before six months, ask why. - How did you learn about that? Did you learn it from someone? - Did you have any problem or challenge (internal or external) impeding the ideal breastfeeding practices? For every problem, probe to talk more about the problem. - What elements do you believe help you to overcome those challenges? Is it something inside you (physical, mental, or spiritual) or something outside yourself (social and physical context)?

During the follow-up interviews, mothers were classified as practicing EBF if they fed their infants only breastmilk from birth (with the exception of prescribed oral medicines and oral rehydration solutions) until 6 months of age. Mothers were classified as not practicing EBF if they fed their infants any non-breast milk liquid or food at one or more junctures since birth in addition to breast milk in the first 6 months. To determine when EBF was terminated, mothers were asked to confirm the baby’s age in months and weeks when foods other than breast milk were initiated.

### Ethical considerations

Ethics clearance for this study was obtained from the Institutional Review Board of the College of Medicine and Health Sciences in Rwanda (Approval notice: No 058/CMHS IRB/2016). All investigators had research ethics training. Permission to collect data in Muhanga was obtained from local authorities. Informed written consent was obtained from every participant for the entire six-month study period prior to participation; however, the ability to leave the study at any point was guaranteed. Participants were assured of confidentiality and the anonymity of recordings, that participation and withdrawal were voluntary, and that gathered information would be used solely for the purpose of the study.

### Analysis

All interviews were audio recorded and transcribed verbatim by the same team. The first author checked the transcripts for quality against the original recordings and against the fieldnotes for accuracy. Interview transcripts were anonymized by using participant codes. At each round of data collection, the first author and another public professional translated 12 transcripts into English to ensure correct coding by English speaking authors. The data were analyzed using Atlas.ti software (version 7.5.10). Thematic data analysis was applied following Braun and Clarke’s protocol [19], which involved the following phases: familiarization with data, generating initial codes, searching for themes, reviewing themes, defining and naming themes, and reporting. The translated transcripts were coded separately by the first and the second author, who then jointly reviewed emerging themes. Thereafter, the first author, who is bilingual, applied the codes and then grouped the codes into major families and themes in English for all the Kinyarwanda transcripts. Following the first author’s coding in English of the Kinyarwanda transcripts, a colleague with experience in qualitative research co-coded 12 of the Kinyarwanda transcripts independently, followed by a discussion and agreement on the different codes. The coding was guided by the research questions on infant feeding intentions, practices, barriers, and facilitators for the first 6 months of each child’s life. Individual researchers’ interpretations and the identified themes were debated and challenged in a series of team meetings involving all authors, from which further analytical refinements emerged. To illustrate themes, typical quotations from participants were translated into English.

## Results

### Characteristics of the study population

Table 2 presents the participants’ characteristics. Their ages ranged from 18 to 40 years, with a mean age of 34 years. Most (82%) were married. The main occupation for all was farming. Ninety-five percent had the ability to read and write their mother tongue. Five percent had not attended any formal education, 49% did not complete primary school, and 38% completed primary school. Seventy-four percent had at least one child, and 77% had some experience of child feeding, including one first-time pregnant mother with experience in child feeding.

**Table 2:** Socio-demographic characteristics of women interviewed

Characteristics, *n* = 39	Frequency	Percentage
Age of the mother (years)		
< 21	4	10%
21–30	13	33%
> 30	22	57%
Marital status		
With partner	32	82%
Ability to read and write		
Yes	37	95%
Education level of the mother		
Illiterate	2	5%
Primary incomplete	19	49%
Primary complete	15	38%
Secondary incomplete	3	8%
Main occupation		
Farming	39	100%
Number of children		
0	10	26%
1–2	21	54%
3+	8	20%
Experience in child feeding		
Yes	30	77%

### Overview of the results

Four major themes emerged representing mothers’ infant feeding intentions and practices during the first 6 months: 1) breastfeeding intentions and reasons behind intentions, 2) breastfeeding practices, 3) barriers impeding early initiation of breastfeeding and EBF for the first 6 months, and 4) facilitators of early initiation of breastfeeding and EBF for the first 6 months. Factors influencing breastfeeding practices during the first 6 months were classified throughout this research at three levels: individual, group, and societal. Tables 3 and 4 summarize the above factors as barriers to, and facilitators of, optimal breastfeeding practices during the first 6 months; these factors are described in more detail below.

**Table 3:** Summary of barriers to breastfeeding practices during the first six months

	Barriers	Intentions	Birth	1 week	4 months	6 months
Individual level factors	Perceived breastmilk insufficiency	×		×	×	×
	Infant health concerns				×	×
	Poor knowledge	×				
	Postnatal discomfort		×			
Group level factors	Family influence			×	×	×
	Domestic conflicts					×
	Use of herbal remedies			×	×	×
Society level factors	Limited professional and social support		×		×	
	Household food insecurity				×	×
	Mothers’ heavy workload				×	×

**Table 4:** Summary of facilitators of breastfeeding practices during the first six months

	Facilitators	Intentions	Birth	1 week	4 months	6 months
Individual level factors	Knowledge about the advantages of breastfeeding within the first hour	×	×			
	Knowledge about the advantages of exclusive breastfeeding for the first six months	×				×
	Confidence in the ability to breastfeed		×			
Group level factors	Social support from family members		×	×		
Society level factors	Support from health professionals	×	×		×	×
	Support from Community Health Workers	×			×	×

#### Breastfeeding intentions and reasons behind intentions

All women (*n* = 39) planned to breastfeed their infants irrespective of the mothers’ socio-demographic characteristics. Of the 39 women, 97% (*n* = 38) intended to breastfeed their infants within the first hour after birth, and 82% (*n* = 32) intended to meet the EBF recommendation for the first 6 months.

The main reasons given by the mothers for their intention to breastfeed the baby immediately after birth included their knowledge of the high nutritional value of colostrum and the immunological protection of the newborn from diseases. For example, one mother said:

*“I will breastfeed the baby within the first hour after birth because the first breastmilk protects the baby against diseases and is very nutritious.”* (W-13)

The mothers appreciated not only the nutritional and immunological benefit of colostrum, but also the mother’s benefits from early suckling because it stimulates breastmilk production. For instance, two mothers said:

*“Colostrum contains all the nutrients the baby needs; it is a problem if the baby fails to get it. In addition, breasting the baby immediately after birth stimulates breastmilk production.”* (W-7)

*“If I recall well for every previous birth, I was encouraged by older mothers to put the baby on breastmilk just to try and stimulate early production of breastmilk. That is the same belief and understanding I still have.”* (W-17)

The emotional bonding of the mother and the newborn was also reported as a factor influencing the intention to breastfeed the baby immediately. For example, one mother said:

*“The source of energy for this is that I am pleased to see my baby putting something in the stomach. Breastfeeding increases our intimacy and the baby feels closer to me as a mother than anyone else. We share life as it used to be during the pregnancy. The increased relationship between me and my baby is the first drive to breastfeed it.”* (W-34)

Knowledge of the recommended EBF duration and its key benefits were identified by some women, including better health and the prevention of diseases like diarrhea that can result from giving other foods. For instance, two interviewed women stated:

*“It is very important to breastfeed the baby exclusively for the first six months in order to protect the baby against diarrhea diseases and the resulting slowed growth, which happens when food is provided before six months.”* (W-24)

*“It is very important to only breastfeed an infant during the first six-month period because mother’s milk contains all the essential nutrients.”* (W-34)

The respondents considered health professionals at health centers, specifically midwives, to be an important source of knowledge to support their intention to breastfeed exclusively. For instance, one mother said:

*“Immediately after delivering, midwives tell you to breastfeed the baby in their presence and you do and when you arrive home you continue following their instructions.”* (W-20)

One young woman expecting her first baby was undecided about how long after birth she would start breastfeeding the baby because of her limited experience in child feeding.

*“It might happen that I do not need to breastfeed immediately after birth for instance as a young and first-time mother that I am, you can understand that I do not know whether I will have to wait either some seconds or hours to initiate breastfeeding.”* (W-06)

Other three mothers did not plan to breastfeed exclusively for the entire first 6 months because they perceived breastmilk insufficiency as a factor that might hinder EBF in that period. These women mentioned that they would not withhold food until 6 months; instead, they would respond positively by providing food for the baby.

*“It happens that the baby at around four months shows strong signs of not getting enough breastmilk. If it ever happens to me, I cannot exclude giving my baby those few fruits and light porridge.”* (W-13)

*“The only obstacle to exclusive breastfeeding that I could foresee is what happens very often for the difficult postpartum breastmilk production, which renders early exclusive breastfeeding very challenging. When I experience that concern there is no other option but to give some half warm milk to the baby on a spoon but after strictly ensuring hygiene.”* (W-24)

*“It may happen that the baby shows interest and eagerness for food even at five months, I will provide to him fruits so that the baby’s growth is not compromised and other food will be given at six months.”* (W-03)

Three mothers did not intend to exclusively breastfeed for 6 months because they perceived diseases and poverty as factors that might impede EBF.

#### Breastfeeding practices

The number of women complying with the recommendations reduced over time: of the 39 women, 34 (87%) were able to initiate breastfeeding within the first hour after birth, 19 (49%) exclusively breastfed their infants for the first 4 months, and 12 mothers (31%) exclusively breastfed their infants for the first 6 months.

#### Barriers impeding optimal breastfeeding practices from birth to 6 months of age

From mothers’ experiences, the following factors impeded EBF:

##### Limited professional and social support

Three women reported not having initiated breastfeeding within the first hour after birth as recommended by the WHO/UNICEF. Mothers stated this was due to receiving limited support from midwives (*n* = 1) and expectant mothers’ escorts (*n* = 2).

Most mothers reported that they received information from health professional staff about EBF for the first 6 months. However, one mother mentioned that she gave her baby a mixture of lemon juice and honey to treat coughing at 3 months of age, advised by the health center staff.

*“When the baby was seriously coughing at 3 months old, I went to the health center, the health professional staff told me to treat this by providing a mixture made with lemon juice and honey, I did and the baby was cured.”* (W-18, month 4)

##### Postnatal discomfort

Two mothers reported that discomfort and feeling tired hindered early initiation of breastfeeding.

##### Perceived breastmilk insufficiency

In the first week after returning home, some women (*n* = 3) reported giving the newborn some liquids, in addition to breastmilk. For instance, one participant reported having introduced cow’s milk to the baby when she was discharged from the health center because of perceived breastmilk insufficiency. The reasons for this perception were no milk being produced when expressing and the baby continuing to cry after feeds. For other mothers (*n* = 10), the perception that breastmilk was insufficient (between 4 and 6 months) was a barrier to EBF to 6 months.

##### Perceived need for traditional medicines

In the first week postpartum, some participants (*n* = 2) reported giving herbal remedies to the baby. The reasons for this perceived need included the baby crying a lot due to stomach pain and the baby not passing stools.

“*Because the baby was crying day and night, when I look back since we arrived home on Friday evening from the health center after delivery, Saturday the child cried all night, but after getting the traditional medicine, the baby stopped crying and slept well*.*”* (W-36, week 1)

“*When we reached home after discharge from the health center, my mother-in-law realized that the baby was not passing stools and then decided to provide herbs. The next day, the baby passed a stool, since then there is no problem.”* (W-26, week 1)

At 4 months postpartum, almost half of the participants (*n* = 15) reported giving herbal remedies (wild leaves) to their children because the infant was crying due to abdominal pain. The following quotation illustrates that:

*“From two weeks after birth, the baby used to vomit after breastfeeding episodes, and I thought she may have a problem related to stomach pain and provided wild leaves. She is now getting better.”* (W-29, month 4)

##### Influence of family members

Female family members were cited as providing barriers to EBF. For instance, one mother reported giving cow’s milk to the child at 3 months, as advised by her older sister.

*“Because the baby used to cry so often during and after breastfeeding episodes, my sister gave him cow’s milk despite the too young age and when she did, the baby was satisfied.”* (W-19, month 4)

Another mother reported giving boiled water to the baby at two and a half months, guided by her mother-in-law.

“*Because the baby was suffering from constipation, my mother-in-law told me to give boiled water, and then I did.”* (W-35, month 4)

##### Perceived infant hunger cues

Between 4 and 6 months, the majority of respondents reported changing their feeding behaviors to introduce other liquids, and in some cases semi-solid food, in addition to breastmilk. Mothers (*n* = 10) stated that, when the baby appeared very interested in family food and keen to eat, they felt they were depriving the infant by withholding food until 6 months and decided to give them food. For example, one mother stated that, on top of having introduced cow’s milk in the first week, she added other foods at 4 months including fruits like banana and tree tomatoes and other foods such as banana plantains, carrots, Irish potatoes and green leafy vegetables.

*“The baby used to cry often while seeing older children eating even during the night and I could spend sleepless nights. I thought the baby was not having enough breastmilk and then decided to feed semi-solid items.”* (W-28, month 4)

##### Mothers’ concerns over their infant’s health issues

For some mothers (*n* = 3), infant illness and weight loss prompted them to interrupt EBF and to introduce early complementary food like cow’s milk in addition to breastmilk before 6 months.

“*I disrupted the exclusive breastfeeding at one week and a half before six months and started giving fruits to the infant because she had been sick and lost weight. When they took measurements and found that the baby had regressed from the previous visit, I decided to give her some fruits before she attained the full six months in order to rehabilitate her*.” (W-15, month 6)

##### Mothers’ heavy workload

Several women (*n* = 9) reported that they could not find enough time to breastfeed and care for their babies because of their many household chores and farm work*.* This was particularly a concern for women between 4 and 6 months postpartum. The following quotations illustrate that point:

*“For instance, if we go for farming and do not have time to come and prepare our lunch, we are bound to having dinner only and therefore one meal a day. In this situation, even the infant does not have that opportunity to be breastfed before we are back from the farm. I personally observe this and I am conscious of the negative effects as regards the infant*.*”* (W-26, month 6)

*“Some of us have to wait until the baby cries to feel the need to breastfeed every time we are busy with household chores or on-farm activities*.*”* (W-5, month 6)

*“Only limited time can hinder doing so. For instance, one can say I am going uphill for animal fodder, and then in the farmland activities once back home, then follow domestic responsibilities like cooking, and all those really pre-empt us from fulfilling our responsibilities as mothers such as entertaining our kids. With that, I cannot mislead you and say that I am up to doing it.” (*W-1, month 4)

##### Household food insecurity

Mothers expressed their concern relating to lack of food in the household due to poverty and the resulting lack of sufficient breastmilk for the baby.

*“Challenges are obvious because, if a mother has gone to bed hungry without food, a baby cannot get any breastmilk to suck. In this case, the only thing the mother can do to content the baby is carry him/her on her back the next day morning until you get some little breastmilk. Otherwise, if you have enough and adequate food as a mother, I do not see anything that can preclude you from breastfeeding your baby as much as possible*.” (W-22, month 6)

##### Conflicts with partner

One woman expressed how conflicts with her partner affect her intimacy with her baby while breastfeeding:

*“It happens that here at home I have disputes with my partner and, in that case, I am completely in a different and unhappy mood. With that distress you would understand that I cannot have any intimacy with my baby while breastfeeding.”* (W-10, month 6)

#### Facilitators of breastfeeding practices from birth to 6 months of age

As well as barriers, women identified facilitators of the successful initiation of breastfeeding within the first hour and continuation of EBF for 6 months:

##### Knowledge about the advantage of early initiation

Knowledge about the advantage of breastfeeding the baby within the first hour after birth was considered to help the mother to initiate breastfeeding at that juncture. For instance, one mother reported being very keen to feed the baby colostrum because she knew its importance.

*“I knew that the child should be breastfeed immediately after birth within 30 minutes, as we were informed during the antenatal education that the first milk that comes is very important for child development.”* (W-29, week 1)

##### Personal confidence in the ability to breastfeed

Confidence in the ability to breastfeed the baby was also reported as a facilitator of early initiation of breastfeeding on top of knowledge that the baby should have breastmilk immediately after birth. For instance, one mother reported:

*“The most important thing is that knowledge of the nutrition value that the baby gets from breastmilk that he/she cannot get anywhere else. Once you have that knowledge and you have your own breast that is all! Again, the first and foremost is the willingness to breastfeed exclusively right from birth.”* (W-34, week 1)

##### Support from health professionals and CHWs

The majority of women considered the practical and informational assistance provided by healthcare professionals from the health center, midwives, and CHWs as particularly important for early initiation of breastfeeding and EBF for 6 months.

*“Immediately after birth, right after I left the delivery room, the midwife told me and was monitoring whether I’m breastfeeding him right and the baby is sucking well.”* (W-21, week 1)

*“We are taught by the health workers. Before we used to give food to children before they reached 6 months of age because, when they saw other people eat, they became greedy and wanted to eat, but today we understand the importance of exclusive breastfeeding for the first six months. We are instructed by health workers, nurses at the Health Centre, and during our gathering for village kitchen activities. Giving hard food to a child before this age is dangerous because its stomach is not yet adapted to this kind of food.”* (W-30, month 6)

##### Social support from family members

Some women cited the support they received from their caretaker (in some instances serving as the expectant mother’s escort) in getting the baby close to them as a facilitator of initiating breastfeeding within the first hour after birth.

*“Because I was not able to sit down and to hold the baby, the caretaker helped me to hold and put the baby on the breast when I left the delivery room.”* (W-07, week 1)

Some other women considered that the support provided by their relatives, including their partners, was very important for their successful breastfeeding and care practices, especially within the first days after birth. The support comprised stepping in to help in performing some household daily duties such as cooking and creating a good environment by providing what was needed by the mother.

*“Also, my husband is helping me in cooking and doing other household duties in these early days after delivery.”* (W-11, week 1)

## Discussion

This study aimed to gain insights into prenatal maternal feeding intentions, the reasons behind these intentions, actual practices, and factors facilitating or impeding recommended infant feeding practices within the first 6 months of a child’s life in Muhanga District, in Rwanda’s Southern Province. Most mothers had the intention of breastfeeding within 1 h and to exclusively breastfeed for 6 months. While most mothers initiated breastfeeding within 1 h of birth, over time EBF declined due to an interplay of barriers.

### Breastfeeding intentions

The majority of participants in our study intended to breastfeed within the first hour after birth and to breastfeed exclusively for the first 6 months. Consistent with other studies in African contexts in Gambia and Ghana [20, 21], our study found that knowledge about the health, emotional, and nutritional value of early initiation and EBF advantages served as a strong motivation for women’s intention to breastfeed. Health professionals, specifically nurses, were said to be an important source of knowledge to support the mother’s intention to breastfeed within the first hour after birth and to continue with EBF for the first 6 months. Our study revealed that 7 out of 39 mothers did not intend to breastfeed exclusively for the entire six-month period because of unsuccessful past experiences of insufficient breastmilk supply. They believed that they would fail again because of their breasts’ anatomical shortcomings.

### Breastfeeding practices

The majority of the interviewed women (*n* = 34) were able to initiate breastfeeding within the first hour after birth. However, 27 mothers reported adding liquids, solid foods, and herbal remedies before 6 months. The RDHS 2014–2015 revealed that 81% of children aged 4–5 months were exclusively breastfed, whereas our study found lower percentages of 49% (*n* = 19) and 31% (*n* = 12) of children exclusively breastfed at 4 and 6 months of age, respectively. This difference may be due to different methodologies used. The RDHS used the 24-h recall method to determine EBF practices. This means that infants who were given other liquids regularly may not have received them in the last 24 h before the survey, and may have led to overestimation of the actual proportion of exclusively breastfed children.

### Barriers to, and facilitators of, breastfeeding practices

Our study has found that women face a number of challenges in maintaining EBF. Perceived breastmilk insufficiency in the quantity needed to meet children’s hunger needs was cited by women as the primary reason to interrupt EBF and introduce other liquids or semi-solid foods. This practice does not follow the WHO recommendations [22]. Researchers cited similar concerns about breastmilk insufficiency as a common reason for early discontinuation of EBF in many other countries, including Kenya [23], Tanzania [24], Zambia [25], South Africa [26], and India [27]. In the present study, the perception of breastmilk insufficiency may be explained by women’s lack of confidence of in their body’s ability to produce adequate breastmilk. Women believed that an infant often crying or taking an interest in others eating were indications that the child was not getting enough from breastmilk and needed additional food. Although according to Gatti these signs might be part of an infant’s feeding cues, they can also be normal infant behavior [28].

In addition, participants linked insufficient breastmilk to poverty-driven food insecurity. Women reported not producing enough food items to cover their needs and having limited financial means to buy food items at the market. Their lived experiences of not having enough food for themselves drives the perception of their body’s inability to produce sufficient breastmilk. Studies of food insecurity in Ghana and Kenya, respectively, suggest that food insecurity undermines maternal confidence, which in turn may negatively affect EBF duration [29, 30]. The impact of food insecurity on EBF needs be further investigated, but efforts to promote EBF in the study setting should consider addressing the issue of mothers’ poverty-driven food insecurity to ensure their good nutritional status and health.

Women’s heavy workload, including daily household duties and farming activities, decreases the amount of time mothers allocate to breastfeeding. This may be explained by limited social support for the mothers, especially from their male partners, in reducing their overload of household chores. Similar results have been found in studies in Rwanda [31], Uganda, and Bangladesh [32, 33]. Efforts to help women reallocate time to feeding and childcare in this context are therefore needed.

During the first 4 months of a child’s life, right from the first week, mothers reported giving their babies herbal remedies believed to treat or provide protection against perceived illnesses such as colic, stomach pains and coughing. Although this practice interferes with EBF, mothers did not consider this as interrupting EBF. This finding is similar to the existing literature, which suggests that the provision of herbal remedies in the first 6 months is a common practice in Sub-Saharan African countries such as Zimbabwe and Ghana [34, 35].

In our study, some mothers reported adding other food, especially between 4 and 6 months, and herbal remedies during the first months of a child’s life, advised by mothers-in-law and older sisters. Similar findings have been found in studies in Nigeria, Ghana, and Zimbabwe [34–36]. Some other mothers reported that their mothers-in-law supported EBF practice. In Ghana, Aguree et al. [37] observed that mothers-in law with appropriate knowledge on the importance of breastfeeding tend to encourage mothers to practice EBF. Therefore, interventions focusing on mothers without considering their family members may fail.

In our study, we found that knowledge about the benefits of early initiation and EBF was a key factor for maternal commitment to breastfeed. Mothers reported receiving knowledge and advice from health professionals on the early initiation of breastfeeding and EBF during antenatal care visits and postnatal periods. These findings are in line with studies in Kenya, Zimbabwe, Ethiopia, and Tanzania, which showed that counselling about breastfeeding during antenatal and postnatal care was significantly associated with EBF for infants less than 6 months old [38–42]. Furthermore, our findings show that CHWs played a critical role in providing health and nutrition education during the postnatal period. Most mothers reported to receive health and nutrition education from CHWs during the community growth monitoring sessions and village cooking demonstrations. This supports the findings from other studies indicating that CHWs play an important role in interventions aiming at improving child survival in community [43, 44]. Regardless of knowledge about the benefits of EBF, there was still a gap between intentions to practice and actually practicing EBF for 6 months. Several other barriers to EBF were found, including perceived breastmilk insufficiency, infant hunger cues, influence of significant others, heavy workload, and the mother’s poverty and food insecurity. Therefore, a lot still needs to be done by health professionals to support mothers in a way that is closer to the everyday situations - the challenges the mothers face - and to help them to find solutions. In addition, mothers could also benefit from support at household level from CHWs to provide individual support, address the challenges mothers face and build their confidence.

At the same time, some women reported their confidence in their ability to breastfeed as a facilitator of EBF. This finding implies that their breastfeeding self-efficacy helped them to persist in the face of, and to overcome, breastfeeding challenges. It is well documented that maternal breastfeeding self-efficacy is a significant predictor of breastfeeding initiation and EBF duration and helps to overcome difficulties encountered in breastfeeding [45, 46].

Socio-demographic factors like maternal age, education, employment, and parity are known to be associated with suboptimal breastfeeding practices in developing countries including Tanzania, Uganda, and Kenya [23, 24, 41, 47]. However, the ways in which these factors influence EBF practice differ in direction from one setting to another. In our study, the variations within the sample and the sample size were too small to explore their influence on breastfeeding practices.

A key strength of this study lies in its longitudinal nature, minimizing the recall bias that may be associated with cross-sectional studies. As the study was limited to one cohort of females, with a minimal sample size (*n* = 39) in Muhanga District of Rwanda only, the generalizability of the findings is low. However, generalizability of the findings was not the main aim. In addition, as data saturation was reached during data collection, the findings were sufficient to provide a deeper description and understanding of infant feeding intentions, actual practices after birth, and factors facilitating or impeding EBF that allow a judgment to be made about the extent to which the findings are relevant and applicable to similar settings. This study provides a view of mothers attending antenatal consultation at health center facilities. It does not provide information on mothers who do not attend antenatal consultations. Thus, the findings of this study may not be representative of EB in the entire community. Furthermore, participants were drawn from the rural Muhanga District. EBF intentions and practices of mothers living in urban settings and with different socioeconomic status should also be further studied. The findings regarding factors that facilitate EBF can be considered a grounded indication of a research phenomenon that merits further attention. It would therefore be interesting for future nutrition promotion research to gain insights into learning mechanisms and coping strategies that play a role in shaping the practices underlying good child growth among mothers who beat the odds in adverse circumstances.

### Through the lens of the TPB

The TPB provides a framework for understanding what influences mothers’ breastfeeding decisions and practices. In this study, mothers’ behavior intention related to their intended behavior regarding practicing early initiation of breastfeeding and EBF for 6 months. The majority of mothers had positive beliefs about both of these recommendations. These beliefs related to high nutritional value of colostrum, immunological protection from disease, prevention of diseases like diarrhea, mother-child bonding, good growth and better health for the baby. Mothers’ beliefs were influenced by their subjective norms, which are based on the perceived social pressure that they feel from their significant others to initiate breastfeeding immediately after birth and to practice EBF for 6 months. Mothers identified social pressure from nurses as an important influence on performing early initiation and EBF for 6 months. Regarding perceived behavior control, a low number of mothers expressed a high level of perceived control. Previous unsuccessful breastfeeding experiences (insufficient breastmilk), disease, and poverty were among the factors that mothers perceived to be out of their control and that they felt they might negatively influence their planned breastfeeding practices.

Given their positive attitude and subjective norms, most participants intended to breastfeed. However, there was a gap between intentions and actual practices. The observed intention- behavior gap could be explained by the fact that the women’s initial intention, assessed during pregnancy, changed throughout the six-month period. Mothers faced a number of challenges that prevented them from performing the behavior they had intended to perform. Challenges included perceived breastmilk insufficiency, belief regarding infant readiness (infant cues), influence of significant others, poverty and food insecurity. These findings confirm that the TPB is not only an effective theoretical framework for predicting breastfeeding behavior, but also a useful tool for guiding breastfeeding interventions. Therefore, interventions to promote EBF should consider helping mothers to develop skills and strategies to deal with the everyday barriers to EBF.

## Conclusion

The aim of this study was to explore prenatal maternal feeding intentions, the reasons behind these intentions, actual practices, and factors that facilitate and impede EBF during the first 6 months of a child’s life in Muhanga District, Rwanda. Most participants intended to breastfeed exclusively for the first 6 months. However, there was a gap between intentions and actual practices. Most mothers were practicing EBF within the first week but EBF gradually declined due to an interplay of barriers at individual, group and societal levels. EBF promotion interventions should consider supporting and equipping breastfeeding mothers with skills to deal with perceived breastmilk insufficiency and to recognize the true signs of baby hunger cues. Furthermore, important influential family and community members should be targeted to encourage mothers to breastfeed. Finally, interventions that consider addressing the issue of poverty-driven food insecurity should not be overlooked either.

### Recommendations

The way health professionals and CHWs support mothers is important and they are doing a good job, but still it is not sufficient. Additional actions are necessary to help mothers successfully overcome the everyday challenges of EBF:

- Educating mothers about the physiology of breastmilk production in order to address beliefs about their breasts’ anatomical shortcomings
- Informing women about how to recognize the true signs of baby hunger cues
- Targeting male partners’ involvement in caregiving and other household responsibilities traditionally performed by mothers
- Involve other important influential family and community members in EBF promotion interventions
- Integrating self-efficacy-enhancing strategies into healthcare professionals’ dialogue with mothers during antenatal and early postpartum periods.

## Data Availability

The data generated and analyzed during the current study are available from the corresponding author on reasonable request.

## Abbreviations

CHWs: Community Health Workers
CMHS: College of Medicine and Health Sciences
EBF: Exclusive Breastfeeding
IRB: Institutional Review Board
RDHS: Rwanda Demographic and Health Survey
TPB: Theory of Planned Behavior
UNICEF: United Nations Children’s Fund
WHO: World Health Organization
